# The Summarized Assessment of Endothelin A Receptor Expression in Renal Transplant Compartments Associated with Antibody-Mediated Rejection

**DOI:** 10.3390/diagnostics11122366

**Published:** 2021-12-15

**Authors:** Mirosław Banasik, Magdalena Kuriata-Kordek, Piotr Donizy, Katarzyna Nowańska, Krzysztof Wiśnicki, Krzysztof Letachowicz, Sławomir Zmonarski, Dorota Kamińska, Oktawia Mazanowska, Tomasz Dawiskiba, Dariusz Janczak, Agnieszka Hałoń, Marta Kepinska, Bartosz Uchmanowicz, Justyna Zachciał, Andrzej Tukiendorf, Magdalena Krajewska

**Affiliations:** 1 Department of Nephrology and Transplantation Medicine, Wroclaw Medical University, 50-556 Wroclaw, Poland; katarzyna.nowanska@umed.wroc.pl (K.N.); krzysztof.wisnicki@student.umw.edu.pl (K.W.); krzysztof.letachowicz@umed.wroc.pl (K.L.); slawomir.zmonarski@umed.wroc.pl (S.Z.); dorota.kaminska@umed.wroc.pl (D.K.); oktawia.mazanowska@umed.wroc.pl (O.M.); magdalena.krajewska@umed.wroc.pl (M.K.); 2 Department of Clinical and Experimental Pathology, Wroclaw Medical University, 50-556 Wroclaw, Poland; piotr.donizy@umed.wroc.pl (P.D.); agnieszka.halon@umed.wroc.pl (A.H.); 3 Department of General, Vascular and Transplant Surgery, Wroclaw Medical University, 50-556 Wroclaw, Poland; tomasz.dawiskiba@umed.wroc.pl (T.D.); dariusz.janczak@umed.wroc.pl (D.J.); 4 Department of Biomedical and Environmental Analyses, Faculty of Pharmacy, Wroclaw Medical University, Borowska 211, 50-556 Wroclaw, Poland; marta.kepinska@umed.wroc.pl; 5 Department of Nervous System Diseases, Faculty of Health Sciences, Wroclaw Medical University, 51-618 Wroclaw, Poland; Bartosz.uchmanowicz@umed.wroc.pl; 6 Department of Clinical Nursing, Division of Nursing in Internal Medicine Procedures, Faculty of Health Sciences, Wroclaw Medical University, 50-556 Wroclaw, Poland; Justyna.zachcial@umed.wroc.pl; 7 Department of Social Medicine, Wroclaw Medical University, 50-556 Wroclaw, Poland; andrzej.tukiendorf@umed.wroc.pl

**Keywords:** endothelin A receptors, non-HLA antibodies, antibody-mediated rejection, allograft injury

## Abstract

The occurrence of anti-endothelin A receptor antibodies may be useful in diagnosis of transplant damage. We noticed that the presence of the endothelin A receptor (ETA receptor) in biopsy compartments is yet to be defined. We decided therefore to analysed the presence and relevance of the ETA receptor in biopsy to define the cause. Our study aims to evaluate the expression of ETA receptors in renal recipients after a biopsy due to the worsening of transplant function. Methods: The expression of ETA receptors was analyzed in renal transplant biopsies using the immunohistochemical method. The evaluation of ETA receptors was performed on paraffin sections. ETA receptor expression was analyzed in four compartments of renal transplant biopsies: glomeruli; vessels; tubular epithelium; and interstitium. The assessment was presented using a three-step scale (0: lack of expression; 1: mild to moderate immunoreactivity; 2: high expression). The results of each compartment from a single biopsy were summarized and assessed in the context of antibody-mediated rejection (AMR). Results: We analyzed 156 patients who had a renal allograft biopsy after renal transplantation. For each patient, we created a summarized ETA receptor expression score. The summarized ETA receptor expression score analysis showed statistically significant differences in patients with and without AMR. In addition, we noticed that patients with AMR had a significantly higher mean summarized expression of ETA receptor score of 3.28 ± 1.56 compared to patients who had a biopsy for other reasons with a mean summarized ETA receptor expression score of 1.47 ± 1.35 (*p* < 0.000001). ROC analysis of the ETA receptor expression score for detecting AMR status showed that the most appropriate cut-off for the test of the chosen binary classifier is between 2 and 3 of the summarized ETA receptor expression score. Conclusions: The expression of endothelin A receptors in renal transplant compartments may be associated with antibody-mediated rejection. The positive ETA receptor staining might be a vital feature in the diagnosis of damage in AMR. The summarized ETA receptor expression score seems to be an exciting diagnostic tool in transplant injury assessment.

## 1. Introduction

Antibody-mediated rejection (AMR) plays a leading role in transplant immunological injury and, consequently, transplant loss, but presently, we know that not only anti-HLA but also non-HLA antibodies may be significant [1,2,3,4,5,6,7,8].

Endothelin A receptor (ETA receptor) is now considered one of the non-HLA antigens, which may be a significant trigger in immunological response and graft loss [9,10,11,12,13].

What is more, the importance of non-HLA response was noted not only in renal recipients but also in patients after liver, heart, lung, intestine, and hand transplantation [14,15,16,17,18,19,20].

The main function of peptides called endothelins (ETs), produced in the endothelium, is vasoconstriction [21]. ETs include peptides described as ET-1, ET-2, ET-3 [22], and ET-1 is a factor that may trigger tubulointerstitial injury and proteinuria when produced in excess by kidneys [23].

We noticed that anti-ETAR antibodies in renal transplant recipients might be associated with a worse transplant function compared to patients without such antibodies [10]. We presented that the expression of ETA receptors in glomeruli might be a feature in the description of injury during AMR [24]. We also described that the expression of ETA receptors in small and intermediate arteries of renal transplants was associated with acute tubular necrosis (ATN) or AMR [25]. Transplant injury induces a humoral response by presenting non-HLA antigenic determinants, which should be protected from circulating antibodies [4]. ETA receptors are antigens that were noted recently and may induce immunization in patients after organ transplantation [14,17,19,26]. Therefore, we determined to evaluate the immunoreactivity of ETA receptors in four compartments of recipient biopsies. The assessment of each compartment was summarized and analyzed in the context of AMR.

## 2. Methods

### 2.1. Participants and Settings

The research involved 156 patients hospitalized from August 2011 to May 2016 in the Clinic of Nephrology and Transplantation Medicine of the University Clinical Hospital in Wroclaw due to renal function deterioration or proteinuria and undergoing renal transplant biopsy following a clinical indication of the standard of care. The indications for biopsy included deterioration of renal function (increase in creatinine of ≥0.3 mg/dL or proteinuria of ≥0.5 g/24 h). The patients were recruited in one center. Written informed consent was collected from all patients. The study was approved by the Wroclaw Medical University Bioethics Committee (KB-300/2018). All methods were applied in accordance with relevant guidelines and regulations.

Characteristics of the patients are presented in Table 1. Tacrolimus or cyclosporine, mycophenolate mofetil, and steroids were used as the initial immunosuppression. Patients with 10–50% PRA or the second transplantation received basiliximab and those with PRA > 50%—thymoglobulin. Cellular rejection was treated as the standard of care with steroids, while AMR was with plasmapheresis, intravenous immunoglobulin (IVIG), and sometimes rituximab or bortezomib. In addition, donor-specific antibodies (DSAs) were tested with solid-phase immunoassay technology (Luminex, Wroclaw, Poland).

**Figure 1:**
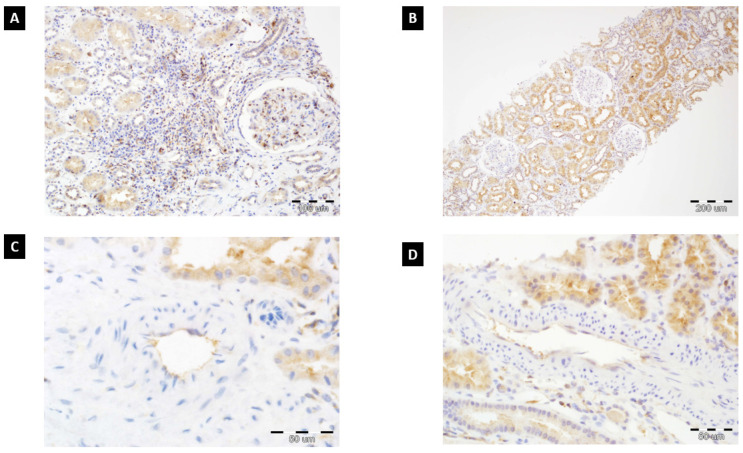
(**A**) High ETAR expression in tubular epithelium, glomerulus, and renal interstitium (chronic inflammatory cells and stromal fibroblasts) (200×, hematoxylin). (**B**) ETAR expression predominantly located in tubular epithelium (100×, hematoxylin). (**C**) ETAR immunoreactivity in endothelial cells of artery with focal expression in tubular epithelium (600×, hematoxylin). (**D**) High ETAR expression in tubular epithelium with immunoreactivity in endothelial cells (400×, hematoxylin).

The expression of ETA receptors was evaluated in renal transplant biopsies with the immunohistochemical method (Figure 1). Microscopic analysis of ETA receptor expression (rabbit polyclonal antibody, dilution: 1:100, catalog number: G094 (P25101); Assay Biotechnology Company, USA) was done on 4 µm thick paraffin sections, which were fitted on silanized slides (DAKO, Denmark). ETA receptor expression was assessed in four compartments of renal transplant biopsies: glomeruli, vessels, tubular epithelium, and interstitium. The results were described using a three-step scale (0: lack of expression, 1: mild to moderate immunoreactivity, 2: high expression). The method and figures were presented and described in our previous paper [24]. In addition, the results of each compartment (0, 1, 2) from a single biopsy were summarized and assessed in the context of AMR.

Renal pathologists (PD and AH) analyzed paraffin sections according to the Banff criteria. Pathologists were not familiar with the DSA status of patients. The presence of C4d deposition was investigated using the immunohistochemical method with a polyclonal antibody.

### 2.2. Data Analysis

Statistica version 13 was used for statistical analysis. A *p*-value below 0.05 was considered significant. The comparisons between baseline predictors and clinical outcomes were carried out using Student’s *t*-test for parametric continuous variables and the Wilcoxon signed-rank test for nonparametric data. Either the Chi-square test or Fisher’s exact test was applied to assess categorical variables. Univariate and multivariate logistic regression analyses were performed to evaluate the association of rejection risk factors for ETA receptor expression. We checked the influence of the number of grafts, recipient age or gender, max PRA, the number of HLA mismatches or anti-HLA antibodies on the presence of ETA receptor expression (Table 2).

## 3. Results

### 3.1. The Summarized ETA Receptor Expression Score

The presence of ETA receptor expression was assessed in four compartments: glomeruli, vessels, tubular epithelium, and interstitium. The analysis was performed in each compartment according to a three-step score where 0: lack of expression, 1: mild to moderate immunoreactivity, and 2: high expression. In the next step, the results from each compartment were added, and the summarized ETA receptor scale was created as in Table 1. No subject scored 7 or 8 (Table 3).

### 3.2. The Summarized ETA Receptor Expression Score and AMR

**Figure 2:**
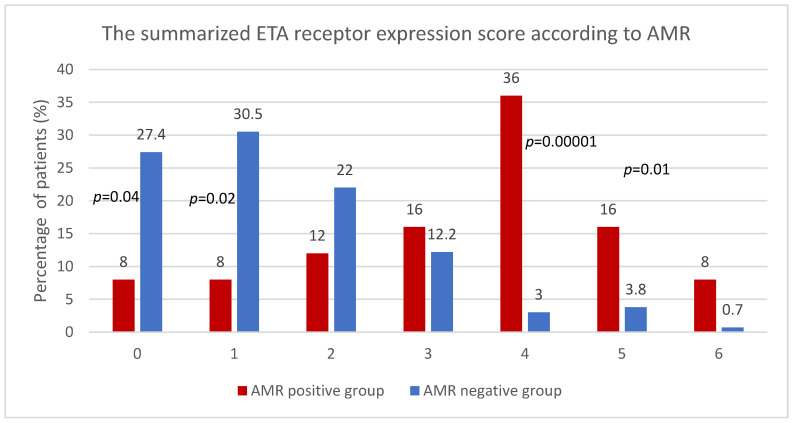
The summarized ETA receptor expression score according to AMR.

The summarized ETA receptor expression score analysis showed statistically significant differences in patients with and without AMR (Figure 2).

### 3.3. The Mean Summarized ETA Receptor Expression in AMR Positive and AMR Negative Group

The group of AMR positive included 25 out of 156 (16%) patients. In addition, patients with an AMR had a significantly higher mean summarized expression of ETA receptor score (3.28 ± 1.56) compared to patients who had a biopsy for other reasons (mean of 1.47 ± 1.35) (*p* < 0.000001).

### 3.4. ROC Analysis of ETAR for Detecting AMR Status

**Figure 3:**
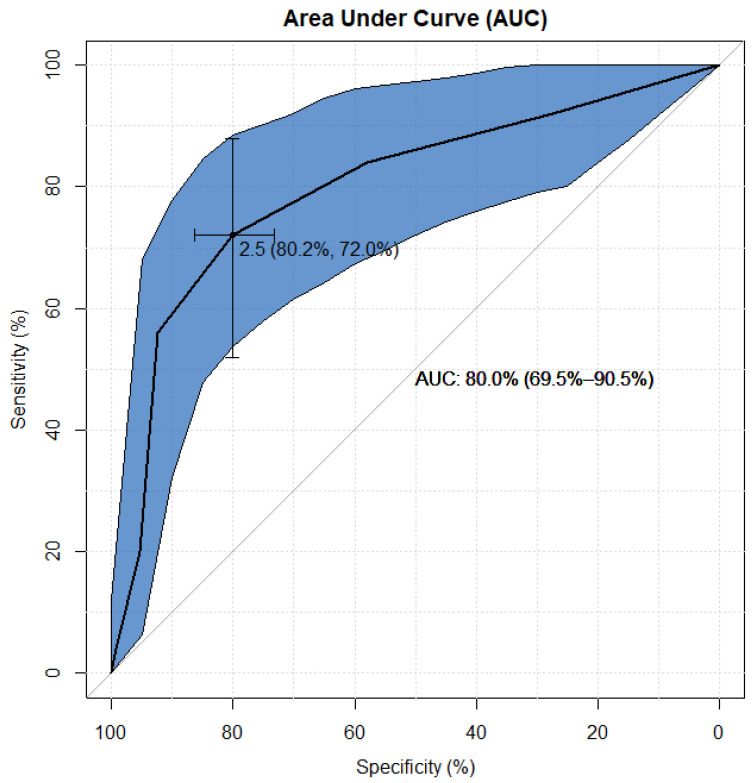
ROC analysis of ETA receptor expression score for detecting AMR status. The most appropriate cut-off for the test of the chosen binary classifier is between 2 and 3 of the summarized ETA receptor expression score at the highest true positive rate, together with the lowest false positive rate, which amount to 72% and 80%, respectively (see Figure 2). Based on the calculated AUC, it can be established that the accuracy of the classification of positive AMR using the level of summarized ETA receptor expression score reached 80% (since the lower limit of the AUC 95% confidence interval is greater than 50%, this result should be considered statistically significant, i.e., *p* > 0.05). Then, it allows to state that 80% of patients would be correctly diagnosed taking only the level of the summarized ETA receptor expression score into account.

In the analysis of the summarized ETA receptor expression score, receiver operating characteristic (ROC) curves were used to choose the most appropriate cut-off for a test of a binary classifier for AMR status [27]. The accuracy of the test was measured by the area under the curve (AUC) and the best cut-off for the highest true positive rate together with the lowest false positive rate. The results of the performed ROC analysis summarized ETA receptor expression score for detecting positive AMR in patients is presented in Figure 3.

## 4. Discussion

We showed that the summarized expression of ETA receptors in the assessed compartments, i.e., glomeruli, vessels, tubular epithelium, and interstitium, might be related to injury described as antibody-mediated rejection. Patients with AMR had a significantly higher mean of summarized ETA receptor expression (3.28 ± 1.56) compared to patients who underwent biopsy for other reasons (1.47 ± 1.35) (*p* < 0.000001). The in-depth analysis showed that patients with AMR had a higher risk of elevated summarized expression than patients without AMR (Figure 1). There is a reverse tendency in patients without AMR—lower expression in more patients with relevant statical significance.

The ROC analysis of the summarized ETA receptor expression score for detecting AMR status suggests that the most appropriate cut-off for the test of the chosen binary classifier is the result between 2 and 3 of the summarized ETA receptor expression score at the highest true positive rate, together with the lowest false positive rate, which are equal to 72% and 80%, respectively.

Based on the calculated AUC, it can be established that the accuracy of the classification of the positive AMR using the level of *summarized ETA receptor expression* score reached 80%. It allows to state that four out of five patients would be correctly diagnosed, taking only the level of the *summarized ETA receptor expression* score into account.

Human leukocyte antigens (HLAs) are known to play a key role in graft loss, but another target called non-HLA antigens (ETA receptors or AT1R receptors) may also be associated with increased occurrence of AMR [5,28]. In 2014, we showed that anti-ETAR antibodies are associated not only with poorer graft function but also with histopathological features characteristic of AMR [10]. Furthermore, anti-ETAR antibodies in this analysis were associated with arteritis and vasculopathy [10].

Our observations inspired us to publish another article showing that the expression of ETA receptors in the glomeruli might be a feature in the diagnosis of damage during AMR [24]. Preformed IgG antibodies targeting non-HLA antigens expressed on glomerular endothelial cells were also described as associated with early acute microvascular rejection [29]. The need for detecting antibodies to HLA but also non-HLA antibodies were noticed by pediatricians to provide a more comprehensive assessment of the patient immune responses to the renal transplant and improved immunological risk stratification [30].

It was presented that autoantibodies comparable to the closely related G-protein-coupled receptor, anti-ETAR, are correlated with anti-AT1R antibodies [13]. Furthermore, we noticed that the expression of AT1R in the tubular epithelium of the biopsy for the cause was associated with significantly higher graft loss [31]. Additionally, the presence of anti-AT1R antibodies in serum, together with the expression of AT1 receptors in transplant biopsies, was also associated with a significantly higher graft loss [32,33].

More and more reports present AMR and graft loss in the absence of donor-specific anti-HLA antibodies. There are increasing suggestions that graft damage is triggered by antibodies against G-protein coupled receptors (GPCR), endothelin A receptor (ETAR), and angiotensin II type 1 receptor (AT1R) [34].

A recently published, remarkable study of 1845 kidney transplant recipients showed at the molecular level the appearance of AMR features in anti-AT1R biopsies associated with increased expression of endothelial-associated transcripts (ENDATs) [5].

We are aware that our analysis of ETA receptors in various compartments and the summarized score of their expression is the first such observation, and further analysis should be carried out to verify our findings. Nevertheless, it seems to be underlined that the expression of ETA receptors may be helpful in AMR diagnosis.

AMR is the most important cause of graft loss.

There is no adequate treatment, so early diagnosis and identification of potential key antigens may be necessary to understand their role in graft injury.

Identification of ETA-mediated lesions may also be useful in potential treatment strategies that prolong graft survival. Additional research is needed to understand the role of endothelin receptors after transplantation.

## 5. Conclusions

The presence of endothelin A receptors in appropriate transplant compartments may suggest a humoral response. Positive expression of the ETA receptor may be a hallmark of graft injury in the progress of antibody-mediated rejection. Therefore, the summarized ETA receptor expression score seems to be an exciting tool in transplant injury assessment.

## Figures and Tables

**Table 1:** Characteristics of the patients according to the presence of AMR.

Patient Characteristics	AMR Positive Group *n* = 25	AMR Negative Group *n* = 131	*p*
Mean summarized expression of ETA receptor score	3.28 ± 1.56	1.47 ± 1.35	<0.000001
Recipient age (years)	41.7 ± 15	43.4 ± 14	NS
Male gender, n (%)	16 (64%)	88 (67.1%)	NS
No. of HLA ABDR mismatches	3.75 ± 0.82	3.56 ± 1.1	NS
A	1.5 ± 0.5	1.32 ± 0.6	NS
B	1.25 ± 0.4	1.35 ± 0.6	NS
DR	0.9 ± 0.4	0.88 ± 0.6	NS
No. (%) of presensitized patients			
max PRA < 10%	17 (68%)	111 (84.7%)	0.0001
max PRA 10–50%	7 (28%)	13 (9.9%)	0.01
max PRA > 50%	1 (4%)	7 (5.3%)	NS
First/next transplantation	24/1	121/10	NS
Cold ischemia time (hours)	22.4 ± 9.2	21.1 ± 9.8	NS
Donor male gender (%)	57%	58%	NS
Donor age (years)	43.7 ± 16.3	50.3 ± 13.2	NS
Cause of chronic renal failure:			
Chronic glomerulonephritis	8	59	NS
Diabetic nephropathy	2	14	NS
Hypertonic nephropathy	3	17	NS
Polycystic kidney disease	3	12	NS
Interstitial nephritis	3	12	NS
Other	6	17	NS
Initial immunosuppression			
Tacrolimus	16 (64%)	92 (70.2%)	NS
Cyclosporin	9 (36%	39 (39.7%)	NS
MMF/MPA	25 (100%)	128 (97.7%)	NS
Azatioprine	0 (0%)	3 (2.3%)	NS
Steroids	25 (100%)	131 (100%)	NS

**Table 2:** Risk factors for ETA receptor expression (in univariate and multivariate analysis).

Analysis:	Univariate	Multivariate
Risk Factor	OR (95% CI), *p*-Value	OR (95% CI), *p*-Value
No. of grafts	0.83 (0.13, 5.52), 0.8483	0.59 (0.06, 5.42), 0.6431
Recipient age	0.97 (0.93, 1.01), 0.1459	0.96 (0.91, 1.00), 0.0621
Male recipient	0.79 (0.24, 2.58), 0.6963	0.73 (0.20, 2.63), 0.6316
Max PRA	0.98 (0.94, 1.02), 0.3506	0.99 (0.95, 1.03), 0.5270
No. of MM HLA ABDR	1.21 (0.85, 1.72), 0.2991	1.10 (0.63, 1.93), 0.7442
Anti-HLA Abs	1.40 (0.44, 4.49), 0.5685	1.46 (0.41, 5.20), 0.5592

OR—odds ratio; CI—confidence interval; PRA—panel reactive antibody; MM—mismatch; No.—number; HLA ABDR—human leukocyte antigen A, B, DR; Abs—antibodies.

**Table 3:** The summarized ETA receptor expression score (*n* = 156).

Summarized ETA Receptor Expression Score	0	1	2	3	4	5	6	7	8
Number of patients	38	42	32	20	13	9	2	0	0
Percentage of patients	24.4	26.9	20.5	12.8	8.3	5.8	1.3	0	0

## Abbreviations

AMR	antibody-mediated rejection:
ATN	acute tubular necrosis;
AT1 receptor	angiotensin II type 1 receptor;
anti-AT1R antibodies	angiotensin type 1-receptor antibodies;
anti-ETAR antibodies	endothelin A receptor antibodies;
DSA	donor-specific antibody;
ET	endothelin;
ERK	extracellular signal-regulated kinase;
ETA receptor	endothelin A receptor;
GPCR	G-protein-coupled receptor;
HLA	human leukocyte antibody;
IVIG	intravenous immunoglobulin;
OR	odds ratio;
PRA	panel reactive antibody.
